# Acceptability and feasibility of early infant male circumcision for HIV prevention in Malawi

**DOI:** 10.1371/journal.pone.0175873

**Published:** 2017-04-17

**Authors:** Charles Chilimampunga, Simeon Lijenje, Judith Sherman, Kelvin Nindi, Webster Mavhu

**Affiliations:** 1 Chancellor College, University of Malawi, Zomba, Malawi; 2 Ministry of Health, Lilongwe, Malawi; 3 UNICEF, Lilongwe, Malawi; 4 Centre for Sexual Health & HIV/AIDS Research (CeSHHAR), Harare, Zimbabwe; 5 Liverpool School of Tropical Medicine, Liverpool, United Kingdom; University of Pittsburgh, UNITED STATES

## Abstract

**Background:**

Voluntary medical male circumcision (VMMC) has been successfully implemented in 14 countries as an additional HIV prevention intervention. As VMMC programs mature in most countries, the focus is now on how to sustain the HIV prevention gains realised from VMMC. As part of preparations for the sustainability phase, countries are either piloting or preparing to pilot early infant male circumcision (EIMC). This qualitative study explored the acceptability and feasibility of EIMC in Malawi in order to inform pilot implementation.

**Methods:**

In 2016, 23 focus group discussions were held across Malawi with participants from several ethnicities and religions/faiths. Additionally, 21 key informant interviews were held with traditional and religious leaders, traditional circumcisers *(ngalibas)*, policy-makers, programme managers and health-care workers. Audio recordings were transcribed, translated into English (where necessary), and thematically coded using NVivo 10.

**Results:**

Discussions highlighted the socio-cultural significance of MC in Malawi. Knowledge or experience of EIMC was poor although acceptability was high among most ethnic/religious groups and key informants. Participants identified EIMC's comparative HIV benefits although a few health-care workers expressed scepticism. All participants said EIMC should be offered within a clinical setting. In addition to fathers, maternal uncles and traditional leaders were deemed key decision-makers. Potential barriers to EIMC included concerns about procedure safety as well as cultural considerations. Key informants felt it was feasible to offer EIMC in Malawi. Participants' recommendations, including phased implementation, engagement of traditional leaders, use of external mobilisers and initially reaching out to influential parents, will be taken into account when designing a pilot EIMC program.

**Conclusions:**

EIMC is potentially an acceptable and feasible HIV prevention intervention for most ethnic/religious groups in Malawi if wide-ranging, culturally appropriate demand-creation activities are developed, piloted, evaluated and appropriately implemented.

## Introduction

Malawi registers approximately 33,000 new HIV infections each year [1]. Although the country's HIV prevalence has declined over the past decade, it remains high at about 10% [1]. Substantially lowering HIV rates in Malawi and the rest of sub-Saharan Africa will only be achieved with the introduction and scale-up of new prevention technologies in combination with existing ones. One key HIV prevention intervention is voluntary medical male circumcision (VMMC), which has been implemented in 14 countries over the last 8 to 9 years. By December 2015, a cumulative total of 11.7 million VMMCs (representing 56% of the target of 20.8 million set in 2011) had been performed in the 14 countries [2]. The 11.7 million VMMCs are projected to avert 335,000 HIV infections by 2025 [2].

Malawi is one of the last countries to scale up VMMC as responsible authorities were sceptical of VMMC's protective effect. The understandable scepticism was due to the fact that the country's 2010 Demographic and Health Survey had included statistics of HIV infection from a sample of 6,834 men, reporting an HIV prevalence of 10.3% in circumcised men, but only 7.9% in non-circumcised men [3–6]. Follow up research established that most men who reported being circumcised were in fact, partially circumcised [3–5]. However, only full MC (complete removal of the foreskin) is protective against HIV. Despite being a later adopter, Malawi has managed to achieve 13% of its set target, and is one of five countries that saw an increase in VMMCs in 2015 compared to 2014 [2].

As VMMC programs mature in most countries, the focus is now on how to sustain the HIV prevention gains realised from VMMC [7–10]. As part of initiatives to prepare for the sustainability phase, pilot implementation of EIMC is already underway in most of the 14 VMMC priority countries including Botswana, Kenya, Lesotho, Rwanda, South Africa, Swaziland, Tanzania, Uganda, Zambia, and Zimbabwe [11–22]. Although its impact on HIV will take longer to realise, EIMC is ultimately likely to be more effective at preventing HIV acquisition than adult MC as the procedure is carried out well before the individual becomes sexually active, avoiding the risk associated with sex during the healing period [23–24]. Like VMMC, EIMC will therefore protect against other sexually transmitted infections and genital cancers in addition to HIV [25–26]. Furthermore, EIMC is cheaper than VMMC, with studies estimating that it is likely to be a cost-saving HIV prevention intervention in the longer term [27–31].

The Malawi Ministry of Health (MoH) has developed an EIMC Roadmap to oversee pilot implementation of EIMC in the country. As part of the Roadmap, the MoH and its implementing partners commissioned a feasibility and acceptability study of EIMC in Malawi. Here we report qualitative findings from the study which will inform the design of a pilot EIMC program.

## Methods

### Design and participants

Between January and March 2016 we collected data using focus group discussions (FGDs) and key informant interviews (KIIs). Previous research showed religion and culture as main determinants of MC in Malawi [3]. The sample for FGDs (n = 23) was therefore based on ethnic and geographical considerations. For FGDs, seven districts were purposively sampled from the country's three regions: Mzimba and Nkhatabay (Northern region); Lilongwe, Ntchisi and Ntcheu (Central region); Mangochi and Mulanje (Southern region, the only traditionally circumcising districts among the sampled districts). Discussions with the Malawi EIMC task force suggested that this sample likely represented most Malawian ethnic groups. The sample size was also deemed sufficient to achieve theme saturation—a situation where qualitative data collection reaches a point where no new constructs emerge [32–34].

FGD participants included: expectant mothers (n = 7 FGDs); expectant fathers (n = 6 FGDs); grandmothers/mothers-in-law (n = 5 FGDs); grandfathers/fathers-in-law/ maternal uncles (n = 5 FGDs). FGD participants belonged to a wide range of ethnicities including: Chewa, Yao, Tumbuka, Lomwe, Sena, Ngoni and Nkhonde. Additionally, some FGD participants identified themselves as either Christian or Muslim. We also held key informant interviews (KIIs) with traditional leaders (n = 3), religious leaders (n = 4), traditional circumcisers (*ngalibas*) (n = 3), policy-makers, programme managers and health-care workers (n = 11). Leaders of traditional and religious groups were purposively sampled to include several ethnicities and religions/faiths. Traditional circumcisers were from Mangochi and Mulanje.

### Data collection, processing and analysis

Although a few key informant interviews were held in English (n = 5), Tumbuka (n = 1) and Yao (n = 2), discussions were mostly in Chichewa, the main indigenous language. Facilitators first explained that EIMC was medically performed during the first 60 days of life and subsequently explored perceptions around its acceptability and feasibility. All discussions were audio-recorded; hand-written notes were taken as back-up. Key informant interviews took approximately 1 hour and FGDs lasted approximately 2 hours.

Data were transcribed and translated verbatim into English. Interview summaries were then written for each KII and FGD and used to come up with a provisional coding framework. Five key informant interviews and four FGDs were then independently coded using the coding framework. Discrepancies were resolved through discussion until consensus was reached. Any additional codes identified from the first set of transcripts were added to the coding framework. Names and other personal identifiers were removed from transcripts before they were entered into NVivo 10 (QSR International, Melbourne, Australia), a qualitative data storage and retrieval program. Transcripts were then coded using the modified coding framework; care was taken to identify any additionally emerging codes. Codes were grouped and emerging themes were then identified.

### Ethical considerations

Ethics approval was given by the Malawi National Health Sciences Research Committee. Written informed consent, including for audio recording, was obtained from all participants prior to their participation in the study.

## Results

A total of 247 participants aged 18–80 years took part in the 23 FGDs. Of these, 133 (54%) were female. In addition, 21 key informants were interviewed. Discussions highlighted the socio-cultural significance of MC in Malawi. EIMC acceptability was high among most ethnic groups although knowledge or experience of EIMC was sub-optimal. Health-care workers felt it was feasible to offer EIMC in Malawi. Participants articulated some concerns and recommendations which should be taken into account when designing a pilot EIMC program. We present these themes in more depth below.

### The socio-cultural significance of male circumcision in Malawi

Despite VMMC roll out in Malawi over the last 5–6 years, male circumcision continues to be associated with religion and ethnicity. Some participants felt that MC was a practice specific to certain ethnic or religious groups. *'*…*We consider it [MC] as something new because here it is only those from Mangochi who get circumcised*. *We*, *the Ngonis and Chewas*, *do not do that'* (expectant mother, Ntcheu). Another female participant maintained, *'Our culture does not allow male circumcision*. *Yes*, *we are not Muslim here*, *it is only Muslims who do that'* (grandmother, Ntchisi).

Terminology used to refer to traditional MC by some groups also mirrors attitudes towards the procedure. *Ongwanjulidwa* is a derogatory term which denotes that one's foreskin has been brutally severed. Conversely, circumcised men use terms such as *osatchena* when referring to an uncircumcised man. Literally, the term implies that one is not smart but the underlying inference is that he is unclean/impure. Among traditionally circumcising groups, there is no terminology that specifically refers to male circumcision since the procedure is part of a comprehensive ‘rites of passage’ ritual; therefore terminology (e.g. *jando*, *chinamwali*, *kuvinidwa*) holistically refers to the entire ritual.

As part of the traditional MC ritual, the initiate is taken to a temporary shelter which is isolated from the community [35]. There, he is circumcised and stays for up to a month as he heals and is taught about manhood according to tradition [35]. Some FGD participants noted that even if MC (and EIMC) is done in a medical setting, it should be complemented by the traditional teachings. *'What I would want to see is that after circumcising the boys at the hospital*, *they should then go to* ***jando***. *Maybe they should go to* ***jando*** *to be taught good manners and then towards the end of the initiation*, *they should be taken to the hospital for male circumcision'* (new mother, Mangochi).

Discussions with young Yao men suggested that the foreskin removal itself was symbolic. *'*…*Removing the foreskin "tells" the boy that he must leave bad habits*…*If they remove the whole foreskin*, *it might suggest that the boy did not have any good manners before and that is why they leave some foreskin'* (new father, Mangochi). Evident in this assertion is the fact that some ethnicities only perform partial as opposed to complete circumcision—a factor which likely explains the discordance between reported male circumcision status and HIV prevalence observed in some Malawian communities [4, 6].

### Acceptability of EIMC

Despite sub-optimal knowledge or experience of EIMC, discussions suggested high willingness to have sons circumcised in most ethnic and religious groups. FGD participants mentioned that they were especially willing to circumcise their grandsons/sons to protect them from possible future HIV infection. *'Since the main aim of male circumcision is to reduce one's chances of contracting HIV*, *I can say infants should be circumcised so as to create a future generation that will have fewer HIV positive people'* (maternal uncle, Lilongwe). Participants felt that EIMC's timing will likely be a crucial factor in protecting males from HIV. *'*…*Some of these men and boys are circumcised when they have already started having sex*. *It will be too late for some of them*. *I mean they will have already contracted HIV'* (expectant mother, Mangochi).

Although EIMC was highly acceptable among younger men in some traditionally circumcising communities, older Yao men expressed some reservations. They noted that the mothers of circumcised infants would need to be involved in the process as they would nurse the wound. *'The women will see and we will be ashamed*. *So in our case*, *we prefer that they are circumcised when they are older*, *when they are 15*, *10 or even 7 years old'* (grandfather, Mangochi). As with other traditionally circumcising communities, allowing women to see (and nurse) the male circumcision wound is considered taboo [36].

Discussions also suggested high acceptance of EIMC among key informants, including both Christian and Muslim leaders. *'I have already said it and I will say it again that after eight days*, *they saw it was proper*…*Even the Lord Jesus was circumcised after eight days*. *So everyone in those days was being circumcised after eight days of birth'* (Christian leader). A Muslim leader also registered his approval. *'According to our religion*, *I don’t see any problem for the infants to be circumcised as long as the parents want them circumcised'* (Muslim leader).

Most health-care workers and policy makers felt that EIMC was a welcome HIV prevention intervention and highlighted HIV-related advantages of EIMC over adult VMMC. *'We are dealing with someone who has neither been nor is sexually active and so the prevention is at least assured unlike in somebody who is already sexually active'* (health-care worker). However, discussions with a few suggested otherwise. *'I am rather sceptical because I know some tribes that perform male circumcision but HIV prevalence is high in those areas*…*'* (policy-maker). Such views are influenced by the discordance between male circumcision status and HIV prevalence in some Malawian communities alluded to earlier. A few health-care workers questioned the rationale of providing EIMC as an HIV prevention intervention. *'Unless there is another reason apart from HIV because if it is about HIV*, *then I think infants are not the best target group*. *It will actually take 15 plus years to see the impact'* (programme manager).

### EIMC provider preferences

All FGD participants who indicated that EIMC was an acceptable intervention felt it should be performed in hospitals, with most preferring doctors as providers. *'I would say doctors*. *They are well-trained and if something goes wrong during the procedure*, *they know what to do'* (new mother, Mangochi). A few participants noted that nurses could perform the procedure as long as they had adequate training. *'It doesn’t matter whether it is a doctor or a nurse*, *as long as they are well-trained to perform it'* (expectant father, Mulanje). Participants also strongly recommended that EIMC should only be done within a clinical setting; they expressed strong reservations with traditional circumcisers. *'*…*The* ***ngalibas*** *might use the same razor blade on all the boys*. *In this era of AIDS*, *it is better to go to the hospital'* (expectant mother, Mangochi).

Contrary to parents' perceptions that traditional circumcisers may also want to be involved in EIMC, all who took part in this study said the procedure was acceptable as long as they were not asked to perform it themselves. *'It [infant] should not be taken to the* ***jando*** *but to the hospital*…*It needs to be breastfed'* (traditional circumciser). Traditional circumcisers were especially unwilling to provide EIMC as the infant's mother would need to be close by as they performed the procedure, which is considered taboo. One traditional circumciser even mentioned that he sends his wife away whenever he needs to perform any male circumcisions from home.

### Early infant male circumcision decision-making

When participants were asked to envisage who would be involved in the process of deciding about EIMC, younger participants (expectant parents) felt that this would involve a discussion between the infant’s parents. Nonetheless, male and female participants of all age groups highlighted the importance of the father in the decision-making process. Younger men from matrilineal communities noted that although they were key decision-makers, they could not make the ultimate decision without consulting maternal uncles. *'It is mostly the fathers after discussing with the boy’s eldest uncle from the mother’s side*…*We cannot really make the decision without consulting the maternal uncles'* (expectant father, Mangochi). In some traditionally circumcising communities, participants felt that since male circumcision is sanctioned by the chief, it would be difficult for them to adopt EIMC without consulting the traditional leader.

### Perceived disadvantages of EIMC

Despite highlighting several advantages of EIMC, participants also articulated possible disadvantages of the procedure, including physical harm. *'I think there are chances that more foreskin than required is removed in infants*… *You look at an infant’s* ***titi*** *(little penis) and all you see is just foreskin'* (grandmother, Lilongwe). As has been found in previous EIMC acceptability studies [24, 36–37], participants were anxious about the fate of the amputated foreskin. *'We do not know what the hospital or the health-care workers will do with the [discarded] foreskins'* (expectant mother, Mangochi). When asked why they were not as concerned with adolescent and adult men's discarded foreskins, one woman responded, *'I think that infants’ foreskins are very tender and so they will be more effective in rituals than boys’ and men’s foreskins'* (expectant mother, Mangochi).

### Key informants' perceptions around feasibility of EIMC

On the whole, key informants felt that it was feasible to introduce EIMC in Malawi. Unlike parents, all health-care workers felt that a wide range of health-care cadres including doctors, clinical officers, nurses and medical assistants could safely perform EIMC with adequate training. Overall, they felt that EIMC was less complicated than some of the procedures they perform. *'Clinical officers and medical assistants are performing major procedures in women and male circumcision is just a minor procedure'* (policy-maker). When asked to give opinions on the facilities that should offer EIMC, most health-care workers noted that it should be offered only at provincial and district hospitals as these already had some operating theatres/rooms and substantial equipment. They however, highlighted the fact that there is a critical shortage of health personnel and the current providers are overstretched.

### FGD participants' recommendations—EIMC introduction

When asked to suggest how to successfully introduce EIMC in Malawi, FGD participants made at least three recommendations: engagement of chiefs, use of external mobilisers and initially reaching out to influential parents. *'They should first discuss with chiefs so that the chiefs are able to understand what it is all about and then they will tell their people that male circumcision is a useful means of protecting male infants and men from HIV'* (expectant father, Mulanje). A female participant also recommended engagement of chiefs in creating EIMC awareness. *'The first important step is for the chief to choose some people who will sensitise the villagers so that they know the benefits of infant male circumcision and what it is all about*…*'* (new mother, Nkatabay). Some participants, however, felt that local mobilisers are often not taken seriously and recommended use of external personnel. *'Mostly*, *those that give out information about male circumcision are from within the village*. *People therefore do not take such information seriously*. *It would therefore be helpful if the various organisations sensitised villagers directly'* (new father, Mulanje).

In addition, participants recommended mobilising influential community members first as they often influence uptake of services. *'I think the first people to take their infants for male circumcision should be people who are influential*, *people who are well-educated so that the rest of us will take on from them'* (expectant mother, Mzimba). Related to this issue, participants mentioned that there is often a 'wait and see' tendency for most new initiatives. *'I think they [parents] would want to first see*…*You know*, *most people wait to see what happens to others*. *So if they see that other people’s infants who have been circumcised are fine*, *they will also have theirs circumcised'* (mother-in-law, Lilongwe).

### Key informants' recommendations

Although health-care workers felt that it was feasible to introduce EIMC in Malawi, they recommended a phased, cautious implementation. *'First of all*, *when you want to implement something*, *you do it on a small scale and then you analyse and see whether or not it works*. *You will then adjust the programme until you offer it on a larger scale'* (health-care worker). Moreover, key informants suggested intensive campaigns to educate parents and the wider community about the procedure. One key informant mentioned that there might be undesirable consequences if women thought, for example, that EIMC was being compulsorily offered. *'Women will refrain from giving birth at the hospital and go to the traditional birth attendants instead'* (traditional leader).

## Discussion

Findings from FGDs and key informant interviews suggest that EIMC is both acceptable and feasible in Malawi. Since some stakeholders (including health-care workers) do not understand the public health rationale behind EIMC, awareness campaigns need to explain the benefits of EIMC on vulnerability to HIV as well as the non-HIV benefits and the advantages of EIMC compared to male circumcision conducted later in life. Moreover, since findings suggest a general lack of knowledge of how EIMC is performed, explaining that the procedure does not require sutures, is characterised by non life-threatening minimal bleeding, easy wound care and faster healing [15–18, 20–21] will likely allay most of the parental concerns that may act as barriers to adoption of EIMC.

The high EIMC hypothetical acceptability found in this study is encouraging but should be interpreted with caution. Studies conducted within the region have shown poor concordance between hypothetical and actual EIMC acceptability. In Zambia and Zimbabwe, actual uptake was lower than previously suggested by hypothetical acceptability studies (11% vs. 97% and 12% vs. 60%, respectively) [15–16, 19, 38–39]. Given the low levels of knowledge or experience of EIMC found in the FGDs and KIIs, it is unclear whether or not the high hypothetical acceptability will translate into actual uptake once EIMC is introduced. It is clear though that participants were very interested in the intervention as described.

FGD and KII findings support the now well-recognised notion that cultural beliefs should be taken into consideration when providing male circumcision [23, 35, 40–41]. FGD findings suggested that some parents had concerns around the removed foreskin. Additionally, some older Yao men opposed EIMC as they felt that it undermines their tradition by allowing women (mothers) to see and nurse the wound, considered taboo. These findings have some implications for both VMMC and EIMC provision. Implementers will need to recognise and understand cultural and religious beliefs attached to male circumcision among certain groups [41]. It will be important to engage key traditional and religious leaders in efforts to mobilise a wider understanding and acceptance of male circumcision for HIV prevention. This is especially so since these were highlighted as key stakeholders. Moreover, engaging traditionally circumcising groups so that they accept medical male circumcision will be important in ensuring that all procedures result in complete foreskin removal. This is especially important since some accounts suggested that some traditional groups perform only partial MC in which case the remaining part remains vulnerable to HIV infection.

Importantly, both EIMC introduction and subsequent roll-out should be done in a manner that does not suggest any compulsion especially since discussions suggested this could possibly result in avoidance of formal health services. A key consideration for introducing EIMC is integration within routine health services for mothers and their children, such as maternal, newborn, and child health (MNCH) services, thereby strengthening these services [9, 42]. Ensuring that EIMC does not in turn, disrupt these services, will be critical.

Key informants raised concerns around shortage of equipment and operating theatres/rooms in health facilities which are not necessarily applicable to EIMC. When EIMC is offered using devices, it requires minimal space (a surgical bed is enough) and equipment [15–18, 20–21]. Although concerns around the shortage of health personnel are understandable, integrating EIMC within MNCH will partly address this challenge. If integrated, EIMC will require routinely performing only a few procedures on newly born males. For example, projections suggest that if EIMC were offered at every health facility in Zimbabwe, and to every newborn male, each facility would only need to perform up to two EIMC per days. Considering that the actual procedure takes an average of 17 minutes [29–30], a provider will ideally need to devote only half an hour to EIMC per day. These projections are similar for Malawi and other VMMC priority countries.

A major strength of this study is that it collected data through a triangulation of FGDs and key informant interviews for a nuanced understanding of the perceived acceptability and feasibility of EIMC in Malawi. There was largely concordance between data obtained through these two methods, highlighting the likely validity of the results and the value of triangulation. Furthermore, the study adds to the growing body of literature on acceptability and feasibility of EIMC. A potential limitation of this study is that it explored EIMC acceptability in the absence of widely-available services or any communication campaign that specifically provides information about EIMC. As stated earlier, hypothetical acceptability may be quite different from actual acceptance when EIMC is eventually rolled-out [13,14,17,38]. It will therefore be crucial to assess EIMC acceptability within the context of actual roll out. Another potential limitation is that although facilitators first explained to participants that EIMC is medically performed, it is possible that some respondents still felt that EIMC would be performed traditionally and therefore needed to conform to norms related to the traditional procedure. Finally, during FGDs researchers did not probe participants' definition of 'doctor' as the term is sometimes used to refer to any male who offers clinical services while females are considered 'nurses'. Future research needs to explore this issue.

In conclusion, study findings suggest that EIMC is potentially an acceptable and feasible intervention in Malawi among most ethnic and religious groups. While findings suggest possible barriers to EIMC, these are potentially surmountable given time. Wide-ranging, culturally appropriate demand-creation activities to promote EIMC need to be developed, piloted and appropriately evaluated in order to support introduction of EIMC in Malawi.
